# Foreign Bodies in the Nose

**Published:** 1901-12-14

**Authors:** 


### FOREIGN BODIES IN THE NOSE.

Mr. F. J. Steward [1] says that all sorts of plans have been described for removing foreign bodies from the nose. One is to insert the nozzle of a politzer bag into the opposite nostril and blow, the idea being to blow the body out through the anterior nares, and in this it may be successful. There is, however, in this plan the serious danger that if the foreign body, together with the swollen mucous membrane around it, should completely block the nostril, one might, in inflating the naso-pharynx, inflate also the middle ear. Now, when a foreign body has been lodged some time in the nose, the post-nasal cavity will probably be infected, and the results of blowing such infected material up the Eustachian tube into the middle ear might be very serious. Another plan is to inject warm lotion either on the same side, so as to wash the foreign body into the naso-pharynx, or on the opposite side, so as to wash it forwards and out of the nostril. There is, however, a risk in sending it backwards, in that it may become impacted in the air-passages. The plan that Mr

Steward recommends, and always uses, and with which he has never had any trouble, depends on the fact that the anterior wall of the nose is a good deal in front of the floor, where the foreign body nearly always lies. "You stand behind the child and take a probe and pass it towards you along the floor of the nose, by which means it is possible to displace the foreign body upwards. Then by using the anterior bony margin of the floor of the nose as a fulcrum, the foreign body may be pushed by the probe upwards and forwards. The foreign body is now situated between two inclined planes which diverge downwards, and are formed by the probe and the anterior wall of the nose, and therefore it can only move in one direction, namely, downwards. Pressure exerted by the probe causes this downward movement, and so the body escapes." All sorts of things have been removed by this method, which is very simple and efficient.

[1] Clinical Jour., Nov. 6.

